# Improving electron transportation and operational lifetime of full color organic light emitting diodes through a “weak hydrogen bonding cage” structure†

**DOI:** 10.1039/d4sc00496e

**Published:** 2024-04-25

**Authors:** Huayi Zhou, Tengyue Li, Mingliang Xie, Yannan Zhou, Qikun Sun, Shi-Tong Zhang, Yujian Zhang, Wenjun Yang, Shanfeng Xue

**Affiliations:** a School of Polymer Science & Engineering, Key Laboratory of Rubber-Plastics of the Ministry of Education, Qingdao University of Science and Technology Qingdao 266042 P. R. China sfxue@qust.edu.cn; b Department of Chemistry Zhejiang Normal University, Key Laboratory of the Ministry of Education for Advanced Catalysis Materials Yingbin Road No. 688 Jinhua 321004 P. R. China sciencezyj@foxmail.com; c State Key Laboratory of Supramolecular Structure and Materials, Institute of Theoretical Chemistry, College of Chemistry Jilin University Changchun 130012 P. R. China stzhang@jlu.edu.cn

## Abstract

Efficient electron-transporting materials (ETMs) are critical to achieving excellent performance of organic light-emitting diodes (OLEDs), yet developing such materials remains a major long-term challenge, particularly ETMs with high electron mobilities (*μ*_ele_s). Herein, we report a short conjugated ETM molecule (PICN) with a dipolar phenanthroimidazole group, which exhibits an electron mobility of up to 1.52 × 10^−4^ cm^2^ (V^−1^ s^−1^). The origin of this high *μ*_ele_ is long-ranged, regulated special cage-like interactions with C–H⋯N radii, which are also favorable for the excellent efficiency stability and operational stability in OLEDs. It is worth noting that the green phosphorescent OLED operation half-lifetimes can reach up to 630 h under unencapsulation, which is 20 times longer than that based on the commonly used commercial ETM TPBi.

## Abstract 2

We report an electron-transporting material with a special long-range ordered “weak hydrogen bonding cage” (PICN), which exhibits high electron mobility. Excellent performances are realized in full color OLEDs with high efficiencies and long operation lifetimes.

## Graphical abstract

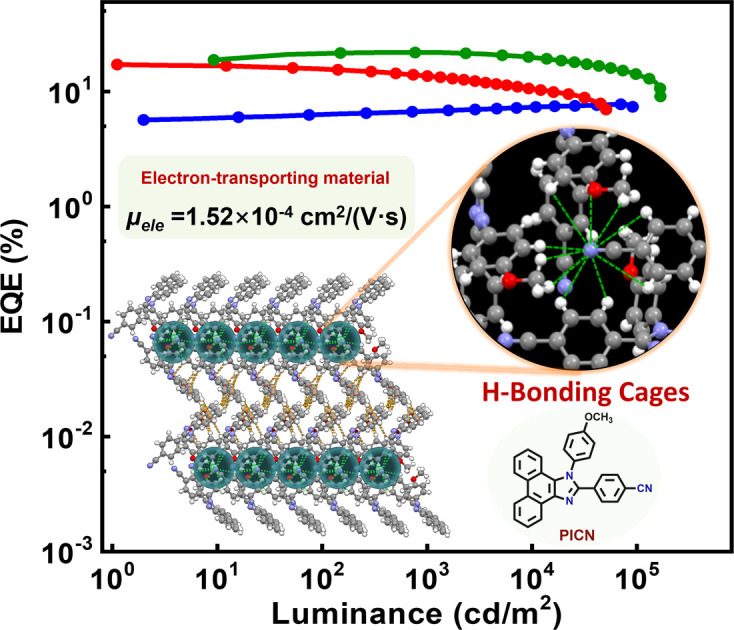

## Introduction

**Scheme 1:**
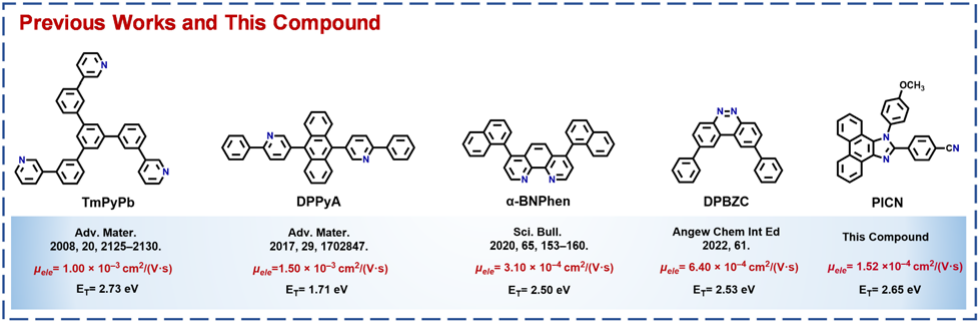
Selected previous excellent ETMs and PICN along with *μ*_ele_s and *E*_T_s.

Organic light-emitting diodes (OLEDs) are gradually gaining shares of the display and lighting markets.^1–5^ Fast and balanced carrier transport in OLEDs has become a significant scientific issue in recent years, which greatly affects the external quantum efficiency (EQE) and operation lifetime of OLEDs.^6–8^ However, the current problem is that the hole mobilities of hole-transporting materials are usually orders of magnitude faster than the electron mobilities (*μ*_ele_s) of electron-transporting materials (ETMs) in OLEDs,^9,10^ which is not conducive to carrier balancing in OLEDs and limits further leaps in OLED device performance to a certain extent. Therefore, the pursuit of ETMs with high *μ*_ele_s has been the key to solving this problem in recent years (*e.g.*, Scheme 1).

In organic semiconductors, electrons are mainly transported between molecules according to a “hopping model”, and therefore, minimizing the distance between adjacent molecules by enhancing intermolecular interactions is crucial to obtain materials with high *μ*_ele_.^11–13^ For example, conventional high-mobility ETMs usually have very large conjugated planes,^14,15^ which depend on strong π–π interactions between molecules to stack molecules tightly, giving the material a high *μ*_ele_. However, such large conjugated planar ETMs also have many problems. First, the surface of the deposited electron transport layer (ETL) is not smooth due to severe π–π stacking,^16,17^ which greatly affects the stability of the device and is not conducive to practical applications. Second, a larger conjugated planar material tends to have a smaller triplet energy level (*E*_T_ ≤ 2.5 eV) and a narrower energy gap (*E*_g_),^18–20^ which easily induce exciton and hole transfer to the ETL, thus degrading the device efficiency and lifetime, particularly for blue and green OLEDs. Moreover, the pursuit of ETMs with large conjugated planar structures encounters demanding difficulties in synthesis and purification,^21^ hindering their further development. Therefore, more unique stacking forms are highly required in obtaining excellent ETMs.

**Scheme 2:**
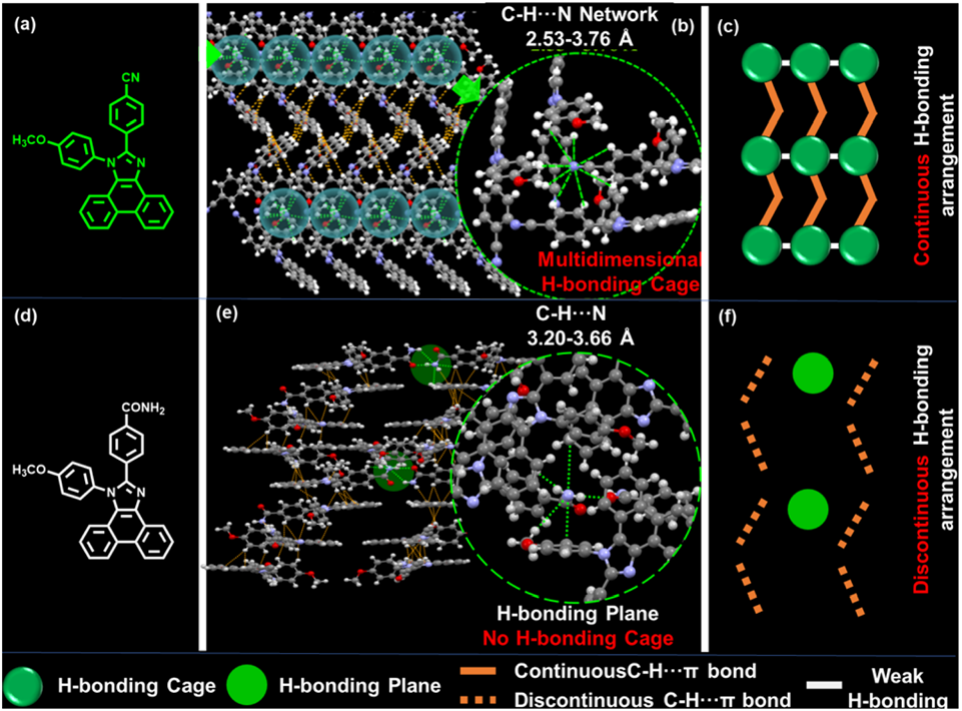
Schematic diagram of the ETMs with three-dimensional hydrogen bonding. (a and d) Molecular structure diagram based on the phenimidazole skeleton. (b and e) Three-dimensional hydrogen bond network structures. (c and f) Intermolecular H-bonding models of PICN and PINH.

In this work, we designed and synthesized a novel ETM (PICNScheme 2a) with phenanthroimidazole (PI) as the backbone. The ETM was created by bonding different heteroatomic groups at the positions of the imidazole ring of PI through a benzene bridge. The single crystal structure of the material PICN reveals that there are massive weak hydrogen bonds, which can form a unique aggregate structure “weak hydrogen bonding cage” (Scheme 2b). This structure is highly ordered, which can act as a fixed button of multiple molecules in the entire crystal arrangement to shorten the intermolecular distance and strengthen intermolecular interactions. As a result, this structure is not only conducive to the enhancement of the electron mobility, but also can significantly enhance material stability and extend the lifetime of OLEDs. Consequently, despite the relatively small molecular weight of PICN, the material exhibited excellent morphological stability. Notably, the three-dimensionally ordered PICN demonstrated a high *μ*_ele_ of 1.52 × 10^−4^ cm^2^ (V^−1^ s^−1^), which is the leading level among reported ETMs.^12,22,23^ Further, the blue fluorescent OLEDs (FOLEDs), green phosphorescent OLEDs (PhOLEDs) and red PhOLEDs using PICN as the ETL all exhibit inspiring performance, with maximum external quantum efficiency (EQE_max_) values of 7.7%, 21.9% and 17.1%, maximum current efficiency (CE_max_) values of 15.7 cd A^−1^, 83.6 cd A^−1^ and 20.6 cd A^−1^ and maximum power efficiency (PE_max_) values of 11.8 lm W^−1^, 89.6 lm W^−1^ and 22.8 lm W^−1^, respectively, accompanied by a low efficiency roll-off (*η*_roll-off_) at high luminance, which is a result of the excellent *μ*_ele_ of PICN. Notably, the OLED operation half-lifetimes (LT50) of the blue FOLED, red PhOLED and green PhOLED are 330 h, 460 h and 630 h under unencapsulation, respectively, which are 4, 8 and 20 times longer than those of TPBi-based OLEDs with the same structure.

## Results and discussion

### Synthesis and structural characterization

The desired molecule PICN was synthesized in one step *via* the Debus–Radziszewski method (shown in ESI†).^24,25^ For comparison, PINH was obtained by hydrolyzing the benzonitrile group of PICN to the benzamide group with more heteroatoms, which has the potential to form richer intermolecular interactions, providing insight into the influence of the strength and arrangement of intermolecular interaction on the properties of ETMs. The yield of the target molecules was approximately 80%. The simple chemical structures make the molecules have good solubility and easy postprocessing. The characteristics and purity of the two molecules were confirmed by ^1^H NMR, ^13^C NMR and mass spectrometry (Fig. S1–S6†).

### Photophysical properties and theoretical calculations

**Fig. 1:**
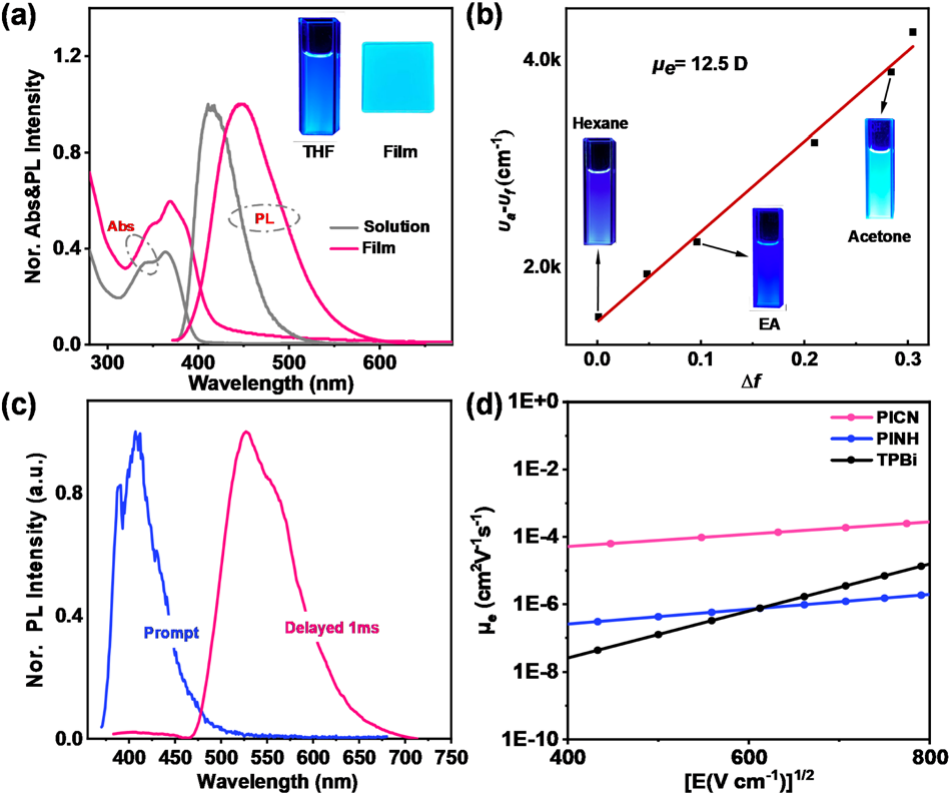
(a) Normalized UV–vis and PL spectra of PICN in a THF solution (10^−5^ M) and the neat film form. (b) Lippert–Mataga solvatochromic model. (c) Fluorescence and phosphorescence spectra of PICN with a delay of 1 ms in 10^−5^ M toluene at 77 K. (d) Electron mobilities of PICN, PINH and TPBi under different electric fields. The configuration of the electron-only device is ITO/LiF (1 nm)/ETM (50 nm)/LiF (1 nm)/Al (100 nm).

The ultraviolet–visible (UV–vis) absorption spectra and photoluminescence (PL) spectra of PICN and PINH in various solvents (10^−5^ M) are depicted in Fig. S7.† With increasing solvent polarity, the absorption spectra of PICN and PINH hardly change in terms of peak position, implying little dipolar variation of the ground state in different solvents. A broad absorption band probably resulting from the intramolecular charge transfer (CT) transition is observed below 400 nm. In striking contrast, their PL spectra exhibit a clear solvatochromic shift in different polar solvents, redshifted by more than 40 nm from *n*-hexane to acetonitrile. As shown in Fig. 1b, the corresponding fluorescence color varies from bright deep blue to sky blue, with photoluminescence quantum yields (PLQYs) of 68.0% and 79.6%. According to the Lippert–Mataga equation, the excited-state dipole moment, *μ*_e_, was calculated to be 12.5 D. Therefore, PICN could be speculated to have typical CT characteristics. Importantly, excitons with CT properties have a weak binding capacity and favor intermolecular electron transfer.^26^ The optimized geometry and electron density distribution of the frontier molecular orbitals (FMOs) were obtained based on the density functional theory (DFT) method of B3LYP/6-31G(d,p) using the Gaussian 09 D.01 package. The twist angle between benzonitrile and PI is as small as 25.7°, causing the benzonitrile and PI moieties to behave as a single large π-bond backbone. As depicted in Fig. S8,† the S_0_–S_1_ transition also exhibits mostly a local excitation (LE) character due to the almost overlapping “hole” and “particle” in natural transition orbitals (NTOs), consistent with the high PLQY of PICN.

The magnitude of the intermolecular interactions can be indicated by the difference between the peak in the PL spectra of the molecules in film form and in solution. Fig. 1a shows that the PL spectrum of PICN in the neat film is red-shifted by 35 nm compared to the emission peaks in solution, whereas PINH in the neat film is red-shifted by 41 nm (Fig. S7†), indicating that the amide group does indeed have richer intermolecular interactions than the cyano group. The phosphorescence and fluorescence spectra of the two compounds at 77 K in N_2_ are displayed in Fig. 1c. The PICN film exhibits two distinct emission features with peaks at 387 and 526 nm originating from prompt fluorescence and phosphorescence, respectively. And the PINH film exhibits emission features with peaks at 401 and 512 nm originating from prompt fluorescence and phosphorescence, respectively. According to calculations made using the starting tangents of the spectra, the *E*_T_s of PICN and PINH are estimated as 2.65 eV and 2.73 eV, respectively (Fig. S9†). These values are close to the ET of TPBi (2.67 eV) and are high enough to prevent the reversed energy transfer of excitons to the ETL.

### Electrochemical and chiroptical properties

The electrochemical behavior of PICN and PINH molecules was studied using the cyclic voltammetry (CV) method. Based on the redox potentials exhibited by the CV curves (Fig. S10†), the HOMO levels of PICN and PINH were calculated to be −5.5 eV and −5.4 eV, respectively, and the LUMO levels were calculated to be −2.6 eV and −2.7 eV, respectively, using ferrocene as a reference, corresponding to *E*_g_s of 2.9 eV and 2.7 eV, respectively. The thermal stabilities of the materials were characterized by thermogravimetric analysis (TGA) and differential scanning calorimetry (DSC). As shown in Fig. S11,† the decomposition temperatures (*T*_d_, corresponding to 5% weight loss) of PICN and PINH were 317 °C and 335 °C, respectively. PICN and PINH have no significant *T*_g_ within the tested temperature range. Furthermore, atomic force microscopy (AFM) images of PICN and PINH illustrated that they both have good morphological stability (Fig. S12†). For the two low-molecular-weight molecules in this work, the excellent thermal and morphological properties may be attributed to the rigid plane of PI and the abundant intermolecular interactions between the molecules.

### Crystallography

**Fig. 2:**
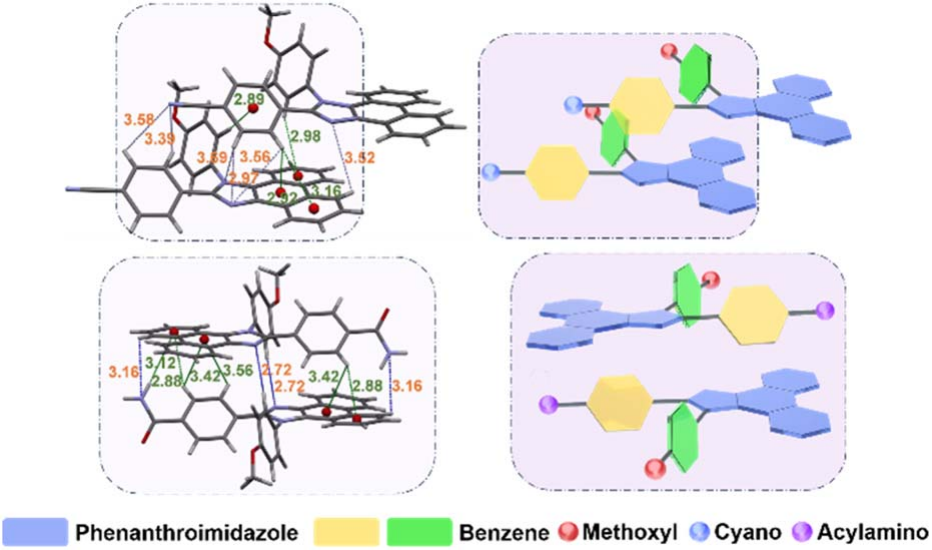
Single-crystal structures of PICN and PINH with bimolecular structures.

The single-crystal structure of the materials was analyzed by X-ray diffraction (XRD) to further understand the details of the intermolecular interactions of the two materials. The crystallography analysis (Fig. S13†) reveals that there is a large torsion angle between the 4-methoxybiphenyl group and the PI group for PICN or PINH, while the group bonded at the C2 position (cyano- or acylamino-) has a smaller torsion angle with PI and forms a conjugated backbone with the PI group, which is consistent with theoretical calculations. The effective π-stacking of the conjugated backbones of two adjacent molecules in the materials is a prerequisite for obtaining high electron mobility. As observed, due to the presence of heteroatoms in the molecules, there are abundant weak hydrogen bonding interactions between the bilayers of both materials, which bring the distance between the two molecules closer. As shown in Fig. S13,† the minimum π-stacking distance between the conjugation planes of both materials is much smaller than the average van der Waals radii (3.5 Å) of two sp^2^-C atoms,^27^ which provides the basic driving force for efficient intermolecular stacking. In multidimensional space, however, the intermolecular interactions between PICN and PINH are not arranged in the same manner. Every four molecules of PICN can form a special “weak hydrogen-bonded cage” structure with the N atom of the –CN as the center and C–H⋯N as its radius (2.53–3.76 Å, Scheme 2 and Fig. 2). The cages possess relatively high combination energy, which promotes stable head-to-head stacking that is favorable for carrier transportation (Fig. S14 and Table S2†).^28,29^ Observed as a whole, the intermolecular interactions of PICN are long-range ordered, thus ensuring an unobstructed electron transport channel.^30,31^ Although PINH has stronger intermolecular interactions due to the substitution of –CONH_2_, the distribution of these interactions is scattered due to the free motion of the groups caused by the excessive flexibility of –CONH_2_, which will cause non-regular C–H⋯N interactions that are not conducive to the electron transition (Scheme 2e and f). Furthermore, the irregular arrangement of PINH increases the possibility of its forming severe traps in amorphous films, which affects the stability of OLEDs.

### Carrier transport properties

The electron-transporting properties of the two materials were investigated using electron-only devices (EODs) with a device structure of ITO/LiF (1 nm)/ETM (50 nm)/LiF (1 nm)/Al (100 nm). The *μ*_ele_s of the target molecules under different electric fields were calculated by the electric field-dependent method. According to the current density (*J*)–voltage (*V*) curves (Fig. S15(a)†), the PICN shows sharply larger current density than PINH in the measured voltage range, and notably, the current density of PICN is also larger than that of the traditional ETM TPBi, indicating its excellent electron transportation ability. As analyzed for the single-crystal structure, PICN with dense and periodic weak hydrogen bonding interactions has good electron transport capacity (*vide infra*) and has an electron mobility of 1.52 × 10^−4^ cm^2^ (V^−1^ s^−1^) at an electric field of 4.4 × 10^5^ V cm^−1^. This is one of the best comprehensive performance results compared to general-purpose commercially available ETMs (*e.g.* Table S3†), and is approximately one order of magnitude higher than that of TPBi. However, the vastly decreased *μ*_ele_ of PINH (9.70 × 10^−7^ cm^2^ (V^−1^ s^−1^)@4.4 × 10^5^ V cm^−1^) compared to that of PICN can be attributed not only to the irregular molecular stacking but also to the fact that –CONH_2_ is not sufficiently stable under an electric field. We have also carried out theoretical calculation on the recombination energies and mobilities using MOMAP 2023 and Gaussian packages with a Monte-Carlo simulation method on the single crystals of PICN and PINH to verify the large difference in the *μ*_ele_ of PINH and PICN (Table S4†).^32–35^ Notably, the calculated electron recombination energy of PINH is as large as >42 eV, while that of PICN is only 0.47 eV. Therefore, the calculated *μ*_ele_ of PINH is near zero, while that of PICN is as high as 0.073 cm^2^ (V^−1^ s^−1^), which is in accordance with the experimental results.

### Electroluminescent devices

**Fig. 3:**
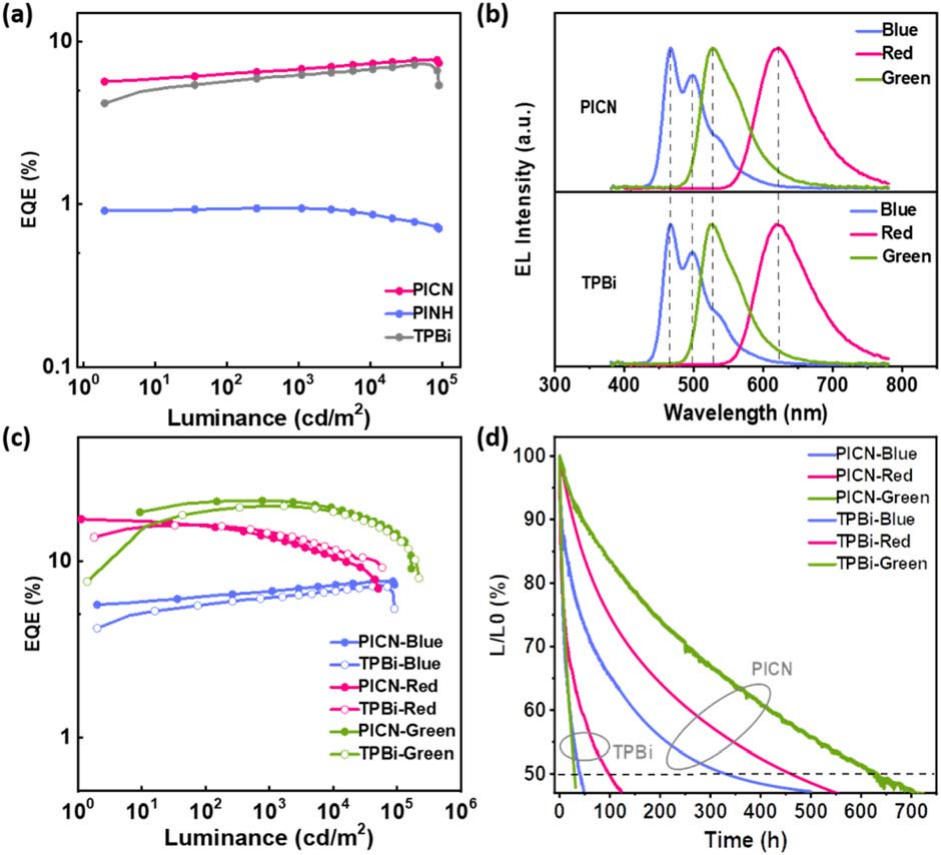
Performances of OLEDs with different ETMs. (a) EQE–*L* curves of the blue FOLEDs based on three different ETMs. (b) EL spectra at 4 V of the devices with PICN and TPBi as the ETMs. (c) EQE–*L* curves of the devices with PICN and TPBi as the ETMs. (d) Lifetime curves of the devices with PICN and TPBi as the ETMs at a fixed current density with an *L*_0_ of 1000 cd m^−2^.

To evaluate the performance of the two materials as ETMs, we fabricated blue FOLEDs with PICN and PINH as ETLs and a device with commercial TPBi as the ETL for reference. The blue FOLED structure is ITO/HATCN (20 nm)/TAPC (40 nm)/TCTA (5 nm)/MADN:DSA-PH (2%) (20 nm)/ETM (40 nm)/LiF (1 nm)/Al (100 nm) (Fig. S16†). The electroluminescence (EL) characteristics of the blue FOLEDs are presented in Fig. 3, S17 and S18,† and the data are summarized in Table S5.† As shown in Fig. 3b, the spectra of the two devices with PICN and TPBi as the ETMs both show single emission from DSA-Ph with peaks at approximately 497 nm and Commission International de L'eclairage (CIE) coordinates of approximately (0.16, 0.31). However, due to the excessively low *μ*_ele_, the spectrum of the PINH-based device showed a shoulder peak near the emission peak at around 433 nm, and its CIE_*y*_ is shifted to 0.20, which indicates that the device has an additional exciton recombination region outside the emitting layer (Fig. S18–S20†). Similar results to those with the EODs are seen at low voltages; however, at high voltages, the current density of TPBi is slightly higher than that of PICN. This difference in current density may be explained by the fact that PICN has a slightly shallower HOMO, which permits some holes to be transferred to the cathode direction and reduces the current density. Among the two novel materials, the PICN-based FOLED achieved a turn-on voltage (*V*_turn-on_) of 3.0 V, a maximum current efficiency (CE_max_) of 15.7 cd A^−1^, a maximum power efficiency (PE_max_) of 11.8 lm W^−1^, a maximum luminance (*L*_max_) of 83 180 cd m^−2^ and a maximum external quantum efficiency (EQE_max_) of up to 7.7%. The PINH-based device shows a *V*_turn-on_ of 3.4 V, a CE_max_ of 1.4 cd A^−1^, a PE_max_ of 1.2 lm W^−1^, a *L*_max_ of 3633 cd m^−2^ and an EQE_max_ of only 0.9%. The comparative device of TPBi exhibits a *V*_turn-on_ of 3.0 V, a CE_max_ of 14.5 cd A^−1^, a PE_max_ of 9.6 lm W^−1^, a *L*_max_ of 91 015 cd m^−2^ and an EQE_max_ of 7.3%, which can be understood by the sharp difference in their *μ*_ele_. Among the OLEDs of the two target molecules, the device performance of PICN is better and achieves a leading level, which can be comparable to the device performance of TPBi. From the analysis of the above data, all devices show low *V*_turn-on_, which is attributed to the suitable LUMO levels of the materials. According to the EQE–*L* curves and CE–*L*–PE curves, the EQEs of the three OLEDs sequentially increase in the order of PINH, TPBi and PICN, which is consistent with the increasing order of *μ*_ele_. This indicates that with the increase in the *μ*_ele_ of the ETM, the carriers in the device tend to be more balanced; thus, the EQE of the device also increases. In addition, due to the high *E*_T_s of the materials, the low-lying triplet excitons (*T*_1_ excitons) are effectively combined in the EL; therefore, the blue FOLED using PICN exhibits very low efficiency roll-off (*η*_roll-off_) and shows excellent stability (no *η*_roll-off_ at a luminance of 60 000 cd m^−2^). The *η*_roll-off_ at high luminance has been reported to be mainly caused by triplet–triplet annihilation and triplet quenching.^36^ Therefore, the strongly restricted *η*_roll-off_ of the OLEDs at a luminance of 60 000 cd m^−2^ can be attributed to the fast utilization of triplet states and the restricted reverse transfer of the *T*_1_ excitons arising from the high *E*_T_s. Furthermore, the performance of PICN, PINH and TPBi in red and green PHOLEDs with a similar structure to the blue FOLEDs was investigated (Fig. S17, S19, S20 and Table S5†). The red and green PhOLEDs based on PICN can also achieve comparable performance to TPBi, showing great potential as a commercial ETM.

Regarding the greatly restricted *η*_roll-off_ and good morphological stability, the lifetimes of the blue FOLED, red and green PhOLEDs with PICN and TPBi as the ETMs were further measured at a fixed current density with an initial luminance (*L*_0_) of 1000 cd m^−2^ under unencapsulation, and the lifetime curves are shown in Fig. 3d. The LT50 lifetimes of TPBi-based blue FOLEDs, red PhOLEDs and green PhOLEDs are 40, 98 and 30 h, respectively. In sharp comparison, the PICN-based devices achieved up to 330, 460 and 630 h of operating lifetime, respectively, which is 4–20 times longer than those of the TPBi-based devices. This can be partially explained by the higher and more balanced electron mobility of PICN-based devices; in addition, the more stable morphological features of PICN also contribute to the long lifetimes (a detailed discussion is in the ESI†). This result is undoubtedly inspiring and proves that PICN is a promising ETM that can contribute to the improvement of device efficiency and lifetime.

## Conclusions

In conclusion, two ETMs with various intermolecular interactions (PICN and PINH) were created by altering the terminal group of C2 of the imidazole ring of PI based on the idea of creating multidimensional hydrogen bonding interactions. In particular, PICN forms a special “weak hydrogen bonding cage” structure between every four molecules with a C–H⋯N radius, which, with the help of C–H⋯N interactions, achieves an outstanding *μ*_ele_ (1.52 × 10^−4^ cm^2^ (V^−1^ s^−1^)) and a high *E*_T_ (2.65 eV). As a result, blue FOLEDs and green and red PhOLEDs fabricated with PICN as the ETL have pleasing device performance, achieving EQE_max_ values of 7.7, 21.9 and 17.1% and remarkable stability (LT50) values of 330, 620 and 460 h, respectively, outperforming devices with the commercial ETM-TPBi as the ETL. The high efficiency and device stability are sufficient to demonstrate the commercial viability of PICN as an ETM. This work is of great significance for designing and synthesizing a kind of electron transport material with excellent comprehensive properties.

## Data availability

Data will be made available on request.

## Author contributions

Huayi Zhou: writing – original draft, writing – review & editing, data curation, conceptualization. Tengyue Li: devices fabrication, validation, data curation. Mingliang Xie: validation, data curation. Yannan Zhou: validation, data curation. Qikun Sun: writing – review & editing, validation. Shi-Tong Zhang: formal analysis, software. Yujian Zhang: writing – review & editing, validation. Wenjun Yang: supervision, investigation, funding acquisition. Shanfeng Xue: writing – review & editing, validation, supervision, investigation, funding acquisition.

## Conflicts of interest

There are no conflicts to declare.

## Supplementary Material

SC-015-D4SC00496E-s001

SC-015-D4SC00496E-s002
