# Supplementary material for: Improving electron transportation and operational lifetime of full color organic light emitting diodes through a “weak hydrogen bonding cage” structure
Source: Chem Sci. 2024 Apr 25;15(21):8106–11. doi: 10.1039/d4sc00496e (PMC11134344; doi:10.1039/d4sc00496e)
Supplement: SC-015-D4SC00496E-s001 [file SC-015-D4SC00496E-s001.pdf]

Electronic Supplementary Material (ESI) for Chemical Science.
This journal is © The Royal Society of Chemistry 2024

SUPPORTING INFORMATION

Table of Contents

Ⅰ. Experimental Procedures 1

Ⅱ. General Methods 2

Ⅲ. Supplementary Results and Discussion 2

Ⅳ. Supplementary Figures 4

Ⅳ. Supplementary Tables 11

Ⅴ. References 13

Ⅰ. Experimental Procedures

1. Synthesis of 4-(1-(4-methoxyphenyl)-1*H*-phenanthro[9,10-*d*]imidazol-2-yl)benzonitrile (PICN)

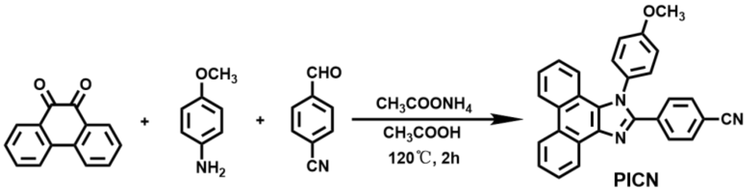

A mixture of 9,10-phenanthraquinone (2.00 g, 9.61 mmol), 4-methoxyaniline (4.73g, 38.4 mmol), 4-formylbenzonitrile (1.26 g, 9.61 mmol), ammonium acetate (3.70 g, 48.03 mmol) and acetic acid (30 ml) was refluxed for 2 h under nitrogen. After cooling down, the solid product was filtrated and washed with 30 ml 1:1 water/acetic acid and 30 mL water successively, dissolved in CH_2Cl_2 and dried in $MgSO_4$, and concentrated in vacuum. It was purified through silica gel column chromatography by dichloromethane (DCM)/petroleum ether (3:1; v/v) to give the product as a cinereus solid. The resulting crude product was purified by recrystallization from ethyl alcohol to give a white solid (PICN, 3.11 g, yield: 76%). ^{1}H NMR (500 MHz, Chloroform-d) δ 8.83 (dd, J = 8.0, 1.5 Hz, 1H), 8.77 (d, J = 8.4 Hz, 1H), 8.70 (d, J = 8.3 Hz, 1H), 7.78 – 7.71 (m, 3H), 7.67 (ddd, J = 8.4, 6.9, 1.5 Hz, 1H), 7.61 – 7.51 (m, 3H), 7.46 – 7.39 (m, 2H), 7.31 (ddd, J = 8.2, 6.9, 1.3 Hz, 1H), 7.27 – 7.22 (m, 1H), 7.16 – 7.10 (m, 2H), 3.98 (s, 3H). ^{13}C NMR (126 MHz, $CDCl_3$) δ 158.71, 146.62, 135.64, 132.94, 129.99, 128.74, 127.91, 127.66, 127.49, 127.00, 126.47, 125.49, 125.06, 124.52, 124.00, 123.43, 122.21, 121.22, 120.94, 120.67, 119.00, 116.61, 113.60, 110.03, 75.31, 75.06, 74.80, 53.74. HRMS (ESI) *m/z*: $[M + H]^+$ calcd for $C_{29}H_{19}N_3O$, 426.1606; found, 426.1599.

2. Synthesis of 4-(1-(4-methoxyphenyl)-1*H*-phenanthro[9,10-*d*]imidazol-2-yl)benzamil (PINH)

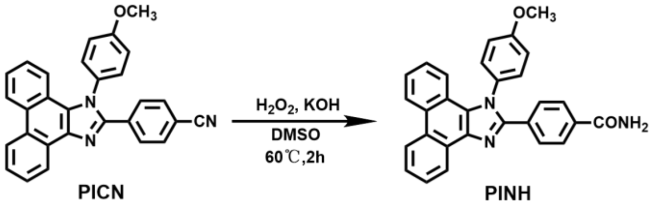

A mixture of 4-(1-(4-methoxyphenyl)-1*H*-phenanthro[9,10-*d*]imidazol-2-yl)benzonitrile (PICN) (1.5 g, 3.52 mmol), 30% H_2O_2 (3.00 g, 88.13 mmol), KOH (1.76 g, 31.37 mmol), and DMSO (30 ml) were stirred for 2 h at 40 °C. After cooling to room temperature, the mixture was extracted with DCM, the combined organic layer dreed with anhydrous $MgSO_4$, filtered and concentrated in vacuo. The crude product was purified by silica-gel column chromatography using ethyl acetate/DCM (3:1; v/v) as the eluent to give the compound as a white solid (1.56 g, Yield 88%). ^{1}H NMR (500 MHz, DMSO-d6) δ 8.93 (d, J = 8.4 Hz, 1H), 8.87 (d, J = 8.4 Hz, 1H), 8.70 (dd, J = 7.9, 1.4 Hz, 1H), 8.01 (s, 1H), 7.88 – 7.83 (m, 2H), 7.78 (t, J = 7.5 Hz, 1H), 7.74 – 7.60 (m, 5H), 7.60 – 7.54 (m, 1H), 7.44 (s, 1H), 7.39 (t, J = 7.7 Hz, 1H), 7.21 (td, J = 6.8, 1.8 Hz, 3H), 3.91 (s, 3H). ^{13}C NMR (126 MHz,

DMSO) δ 167.18, 160.06, 149.93, 136.43, 134.20, 132.81, 130.40, 130.19, 128.75, 128.56, 128.14, 127.67, 127.44, 127.28, 126.70, 125.76, 125.30, 124.44, 123.63, 122.51, 121.96, 120.26, 115.36, 55.56, 39.99, 39.82, 39.66, 39.49, 39.32, 39.16, 38.99. HRMS (ESI) *m/z*: $[M + H]^+$ calcd for $C_{29}H_{21}N_3O_2$, 444.1712; found, 444.1704.

Ⅱ. General Methods

Prior to the measurement, all of the two target molecules were obtained as pure products by vacuum sublimation. TPBi used in the device was purchased from Jilin OLED Material Tech Co., Ltd., and HATCN, TAPC, TCTA, $Bepp_2$ and MADN were purchased from Xi'an Polymer Light Technology Corp. The optimized geometry and electron density distribution of the frontier molecular orbitals (FMOs) were obtained based on density functional theory (DFT) method of B3LYP/6-31G (d, p) using the Gaussian 09 package. Thermal gravimetric analysis (TGA) of the materials was performed using a Perkin-Elmer thermal analysis system at a ramp rate of 10 °C/min under nitrogen atmosphere. The differential scanning calorimetry (DSC) data of the materials in the range of -50 to 350 °C were tested under a heating rate of 10 °C/min using NETZSCH (DSC-204) instrument. In a nitrogen-purged dichloromethane (DCM) or N,N-Dimethylformamide (DMF) solution at room temperature, the Cyclic voltammetry (CV) analysis was measured. All the potentials with ferrocene/ferrocene + (Fc/Fc +) as standard, and tetra-n-butyl-ammonium hexafluorophosphate (TBAPF6, 0.1 m in acetonitrile) as the supporting electrolyte.The Ultraviolet Visible (UV–vis) absorption spectra of the solutions and films were scanned with a Hitachi U-4100 spectrophotometer. The photoluminescence (PL) spectra of the solutions and films were recorded with a Hitachi U-4600 spectrometer. Single crystal x-ray diffraction data were acquired by a Rigaku RAXIS-PRID diffractometer. ITO patterned anode with a sheet resistance of 5~20 Ω/square was used as the substrate. Before passing into a deposition chamber, the ITO substrate was scrubbed with Hellmanex™ III, followed by ultrasonic treatment with acetone solvent, Hellmanex™ III, and deionized water for 15 minutes each, dried at 120 °C in an oven. The clean ITO substrate is surface oxygenated and subsequently passed into the vacuum vapor deposition bin. The cathode layers and organic layers were deposited in different chambers with a base pressure of less than 1.6×10^{-4} Pa. The single-electron devices of the ETMs were fabricated with the structure of ITO/LiF (1 nm)/TPBi (10 nm)/ETMs (50 nm)/LiF (1 nm)/Al (100 nm), and the single-hole devices of the ETMs were fabricated with the structure of ITO/HATCN (20 nm)/ETMs (50 nm)/HATCN (20 nm)/Al (100 nm), in which lithium fluoride (LiF) has the function of electron injection and hole blocking, while 2,3,6,7,10,11-hexacyano-1,4,5,8,9,12-hexaazatriphe-nylene (HATCN) has the function of hole injection and electron blocking. The OLEDs of the ETMs were fabricated the structure of HATCN (20 nm)/ TAPC (40 nm)/ TCTA (5 nm)/ ELMs (20 nm)/ ETMs (40 nm)/ LiF (1 nm)/ Al (100 nm), where 2,3,6,7,10,11-hexacyano-1,4,5,8,9,12-hexaazatriphe-nylene (HATCN) used as a hole-injecting layer, 4,4′-Cyclohexylidenebis[N,N-bis(4-methylphenyl)benzenamine] (TAPC) used as a hole transport layer, thicker 4,4′,4″-tri-9-carbazolytriphenylamine (TCTA) was used as a electron blocking layer, the LiF served as the electron-injecting layer. For emitting materials (ELM), MADN:DSA-PH (2 %) for blue FOLEDs, $BePP_2$: $Ir(MDQ)_2(acac)$ (15 %) for red PhOLEDs, and $BePP_2$: $Ir(ppy)_2(acac)$ (15 %) for green PhOLEDs. The current density (J), voltage (V), and luminescence (L) characteristics of OLEDs were acquired using a Keithley source measurement unit (Keithley 2450 and LS-160), electroluminescence (EL) spectra were measured with Flame-S (Serial Number: FLMS16791, Range: >350 nm).

The EQEs were calculated from the L, J and EL spectra. The calculation formula (**Formula S1**) is as follows:

$$EQE = \frac{\pi \cdot L \cdot e}{683 \cdot I \cdot h \cdot c} \cdot \frac{\int_{380}^{780} I(\lambda)\cdot\lambda\cdot d\lambda}{\int_{380}^{780} I(\lambda)\cdot K(\lambda)\cdot d\lambda} \qquad \text{(Formula S1)}$$

where L (cd m^{-2}) is the total luminance of the device, I (A) is the current, λ (nm) is EL wavelength, I(λ) is the relative EL intensity at each wavelength and is obtained by measuring the EL spectrum, K(λ) is the Commision International de L'Eclairage chromaticity (CIE) standard photopic efficiency function, e is the charge of an electron, h is the Planck's constant, c is the velocity of light. All the above data were measured in the forward-viewing direction without using any light out-coupling technique.

The efficiency roll-offs were calculated from maximum EQE (EQE_{max}) and the EQE at luminescence of 1000 cd m^{-2} (EQE_{1000}). According to equation following (**Formula S2**):

$$\eta_{roll-off} = \frac{EQE\,max - EQE1000}{EQEmax} \qquad \text{(Formula S2)}$$

Ⅲ. Supplementary Results and Discussion

We prepared green PhOLEDs with $Bepp_2$ as the electron transport layer (ETL) as an example to analyze the reason for the long lifetime of OLEDs with **PICN** as the ETL. Firstly, we found that $Bepp_2$-based green PhOLEDs also have a long lifetime of up to 526 h (**Figure S21**), which is much higher than the lifetime of OLED devices when TPBi is used as the ETL (30 h), but does not reach the lifetime of OLED devices when **PICN** is used as the ETL (630 h). Also, $Bepp_2$ (10^{-4} $cm^2/(V·s)$) and **PICN** (1.52×10^{-4} $cm^2/(V·s)$) have higher μ_{ele}s than TPBi (1.68×10^{-6} $cm^2/(V·s)$) and provide more balanced carrier mobility when prepared into devices, which perhaps largely avoids polariton-exciton collisional bursts, thus providing a more stable luminescence environment and enabling longer device lifetimes when $Bepp_2$ and **PICN** are used as ETLs. In addition, although $Bepp_2$ and **PICN** have similar μ_{ele}, longer lifetimes exist for devices using **PICN**, so we believe that carrier balance is one of the factors for this long lifetime. In order to reveal more fully the differences in the lifetimes of TPBi and **PICN** OLEDs, we vaporised pure films of the two materials separately and explored their morphological stability by atomic force microscopy (AFM). As shown in **Figure S13**, the AFM result and the root-mean-square (RMS) value of the freshly vapour-deposited **PICN** film and the AFM result and RMS value of the film after 96 h in the glove box were tested, respectively. When the films are freshly prepared, they all show very smooth surface, with the root-RMS in the order of **PICN** (2.35 nm) < TPBi (2.65 nm). In addition, DSC was tested in the temperature range of 25~300 ℃, and it was found that **PICN** has no obvious glass transition temperature, and there is no cold crystallization peak during the secondary heating process, which also indicates that the material has good morphological stability. Thus the morphology of **PICN** only show a little change when placed in the glove box for 96 h, while the deformation of TPBi (RMS=4.27 nm) is much more severe than that of **PICN**, which would be harmful for devices by causing a severe interface separation. In summary, the long lifetime of **PICN** is caused by a more balanced carrier mobility and a more stable morphology.

Ⅳ. Supplementary Figures

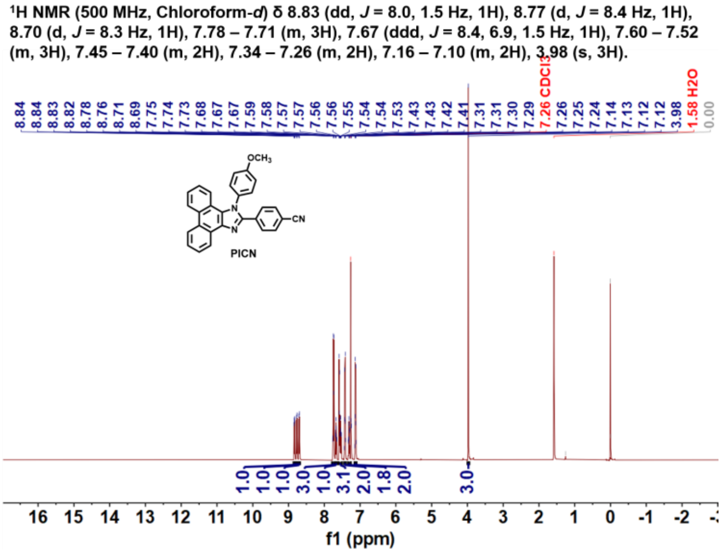

Figure S1. ^{1}H-NMR Spectrum of PICN in $CDCl_3$.

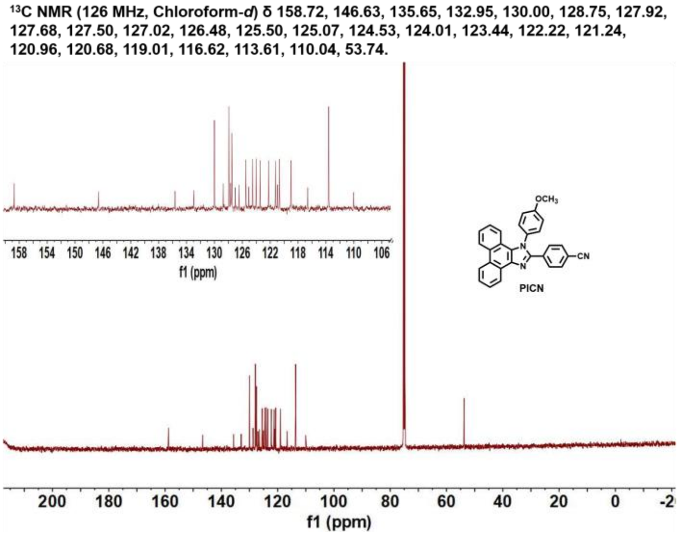

Figure S2. ^{13}C-NMR Spectrum of PICN in $CDCl_3$.

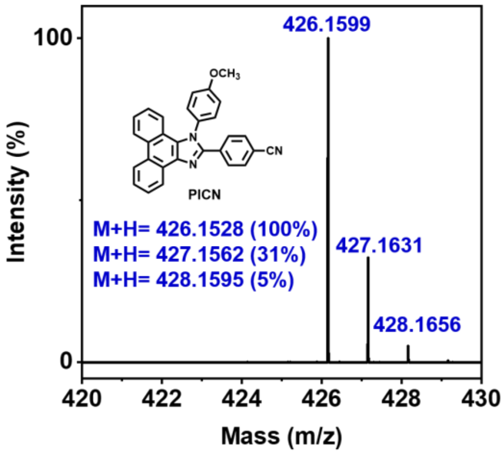

Figure S3. Mass Spectrum (M+H+) of PICN.

^{1}H NMR (500 MHz, DMSO-d_6) δ 8.93 (d, *J* = 8.4 Hz, 1H), 8.87 (d, *J* = 8.4 Hz, 1H), 8.70 (dd, *J* = 7.9, 1.4 Hz, 1H), 8.01 (s, 1H), 7.88 – 7.83 (m, 2H), 7.78 (t, *J* = 7.5 Hz, 1H), 7.74 – 7.61 (m, 5H), 7.61 – 7.54 (m, 1H), 7.46 – 7.36 (m, 2H), 7.21 (td, *J* = 6.8, 1.8 Hz, 3H), 3.91 (s, 3H).

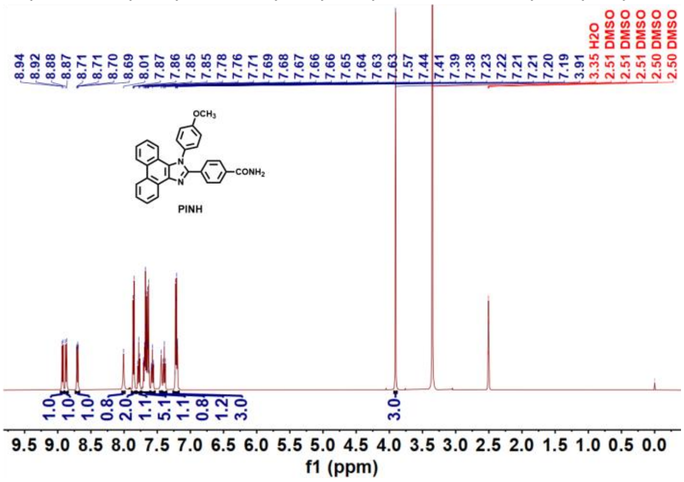

Figure S4. ^{1}H-NMR Spectrum of PINH in DMSO.

^{13}C NMR (126 MHz, DMSO) δ 167.18, 160.06, 149.93, 136.43, 134.20, 132.81, 130.40, 130.19, 128.75, 128.56, 128.14, 127.67, 127.44, 127.28, 126.70, 125.76, 125.30, 124.44, 123.63, 122.51, 121.96, 120.26, 115.36, 55.56, 39.99, 39.82, 39.66, 39.49, 39.32, 39.16, 38.99.

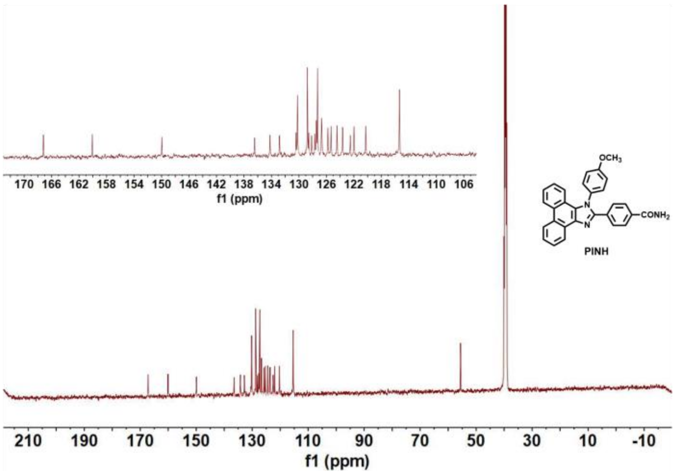

Figure S5. ^{13}C-NMR Spectrum of PINH in $CDCl_3$.

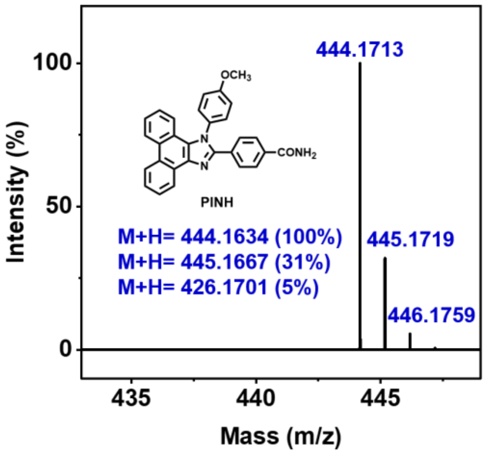

Figure S6. High-resolution Mass Spectrum (M^+H^+) of PINH.

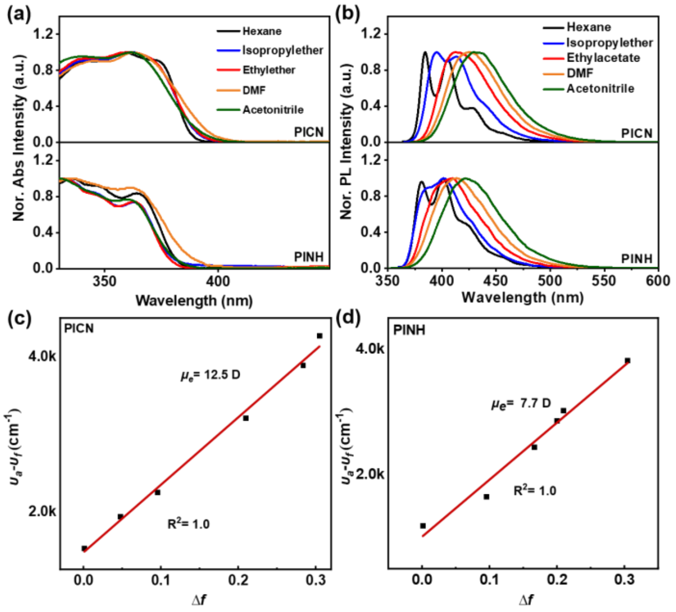

Figure S7. (a) Normalized UV–vis absorption spectra of the two molecules in Different Polar Solvents. (b) Normalized PL spectra of the two molecules in Different Polar Solvents. (c) and (d) Lippert-Mataga solvatochromic model of the two molecules.

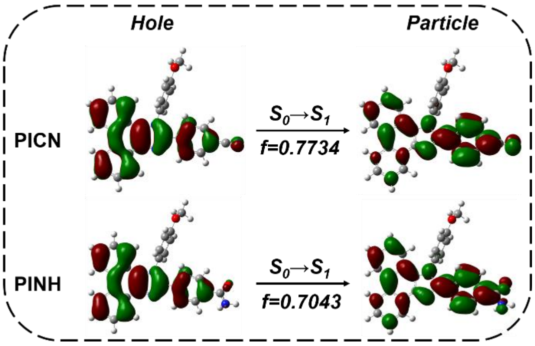

Figure S8. The NTOs of $S_0 \rightarrow S_1$ for PICN and PINH.

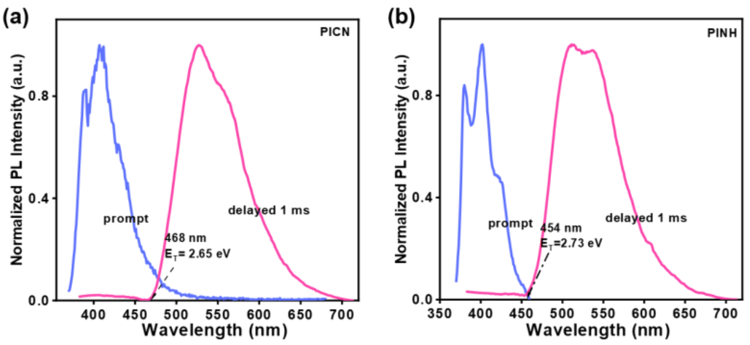

Figure S9. Fluorescence and phosphorescence spectra of the two molecules in 10^{-5} M toluene at 77K.

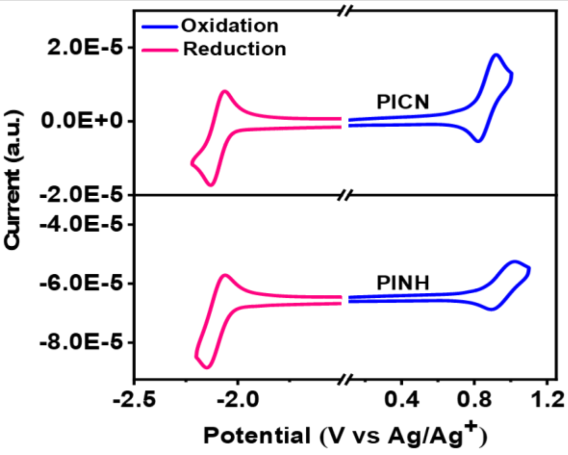

Figure S10. The cyclic voltammetry (CV) curves of the two molecules in DCM solution (oxidation section) and DMF solution (reduction section).

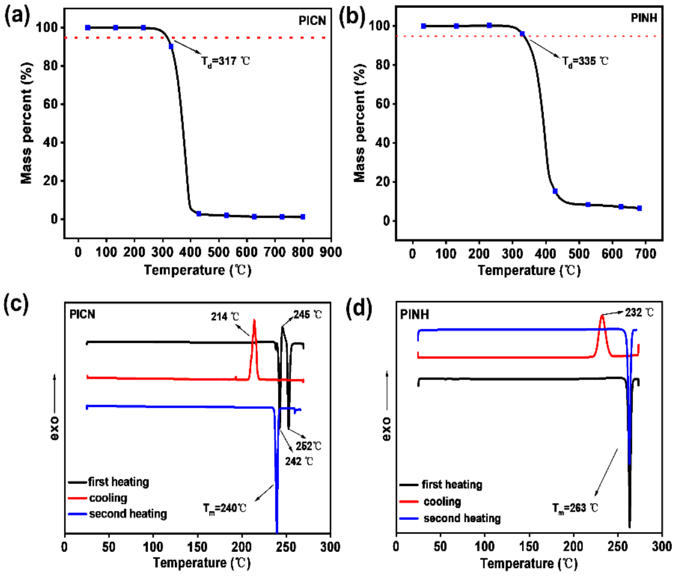

Figure S11. (a) and (b) are the thermogravimetric analysis (TGA) curves of the two molecules. (c) and (d) are the differential scanning calorimetry (DSC) curves of the two molecules.

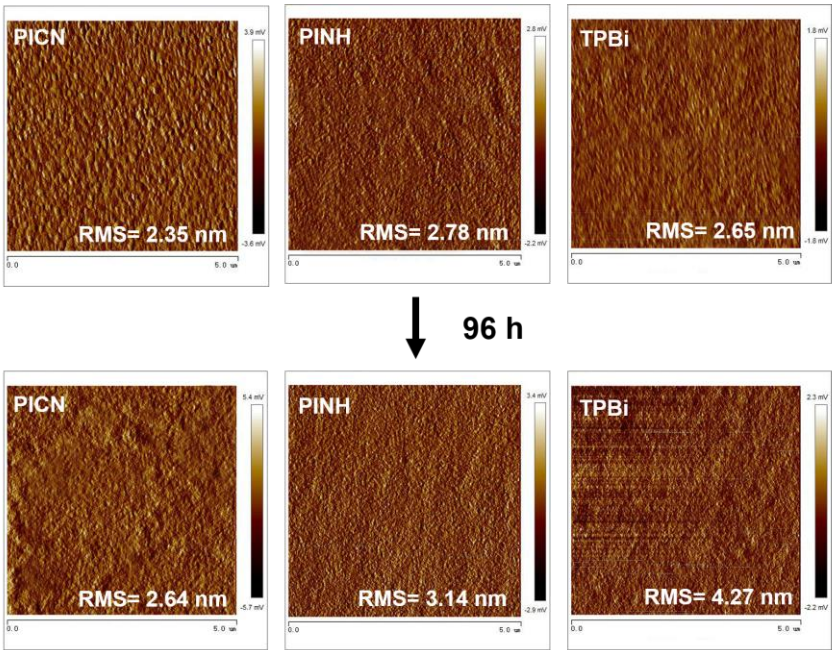

Figure S12. The morphology of **PICN**, **PINH** and TPBi films measured right after and 96 h in the glove box after the film preparation.

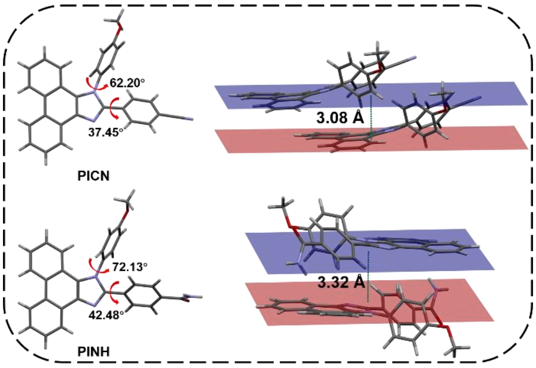

Figure S13. (a) PICN and (b) PINH single crystal structures with torsion angles and minimum distance diagram of the conjugate planes.

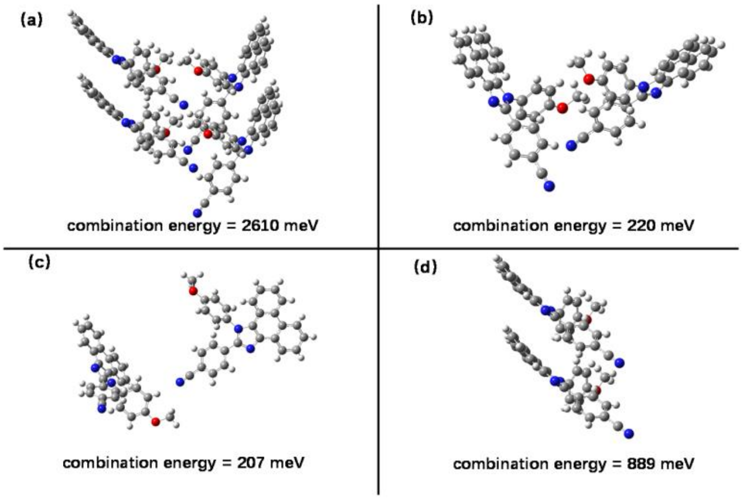

Figure S14. Estimation of the combination energy of the weak hydrogen bond cage of **PICN**. The calculation method is b3lyp/6-31g(d,p) considering the basis set superposition error (BSSE) correction and dispersion correction (GD3).

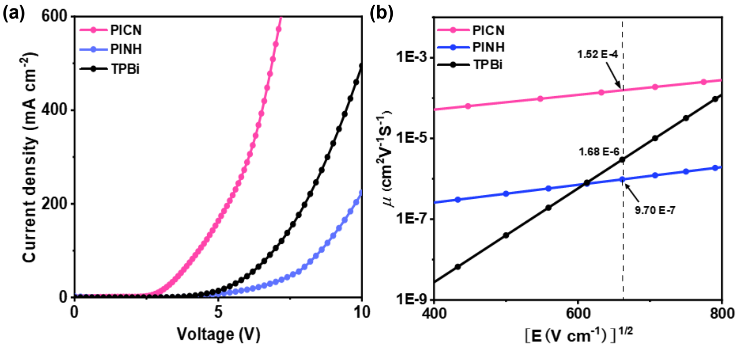

Figure S15. (a) Electron-only J-V curves, (b) Electron mobilities of **PICN**, **PINH** and TPBi under different electric fields.

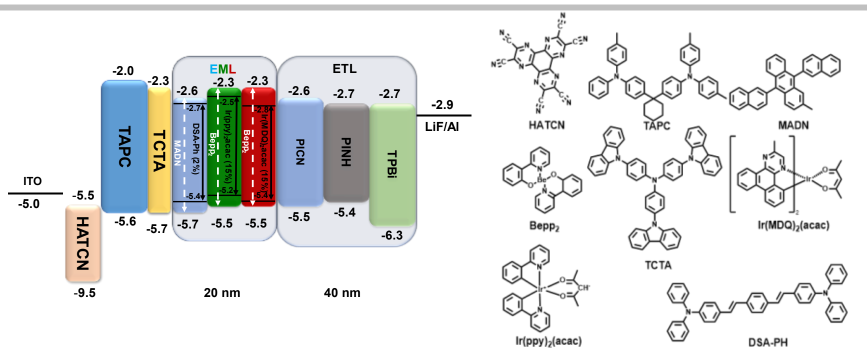

Figure S16. Devices architecture, energy diagram, and functional layers for the vacuum-deposited OLEDs. The blue FOLED structure is: ITO/HATCN (20 nm)/TAPC (40 nm)/TCTA (5 nm)/MADN:DSA-PH (2 %) (20 nm)/ETMs (40 nm)/LiF (1 nm)/Al (100 nm). The red and green PhOLEDs' structures are: ITO/HATCN (20 nm)/TAPC (40 nm)/ TCTA (5 nm)/BePP$_2$:Ir(MDQ)$_2$(acac) (15 %) (20 nm)/ETMs (40 nm)/LiF (1 nm)/Al (100 nm) and ITO/HATCN (20 nm)/TAPC (40 nm)/TCTA (5 nm)/BePP$_2$: Ir(ppy)$_2$(acac) (15 %) (20 nm)/ ETMs (40 nm)/LiF (1 nm)/Al (100 nm).

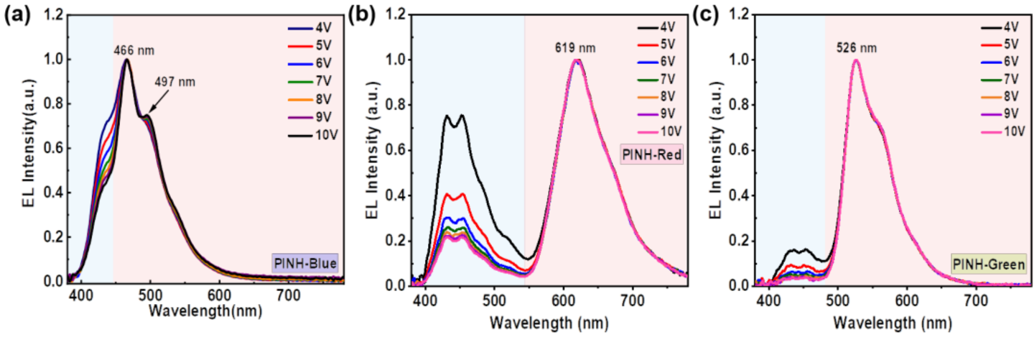

Figure S17. Normalized EL spectra of PINH at different voltages.

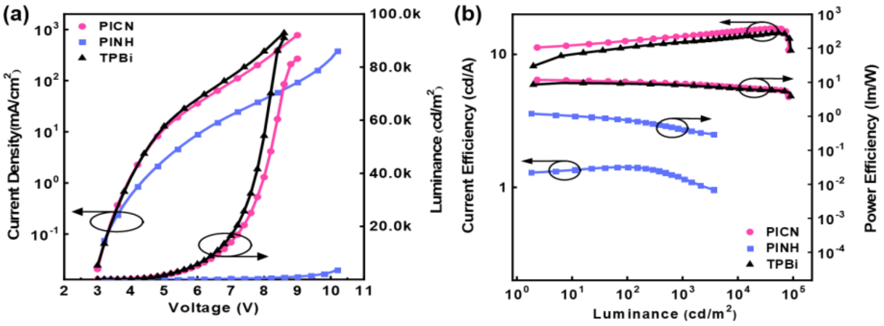

Figure S18. Performances of blue FOLEDs of different ETMs. (a) J-V-L curves, (b) CE-L-PE curves.

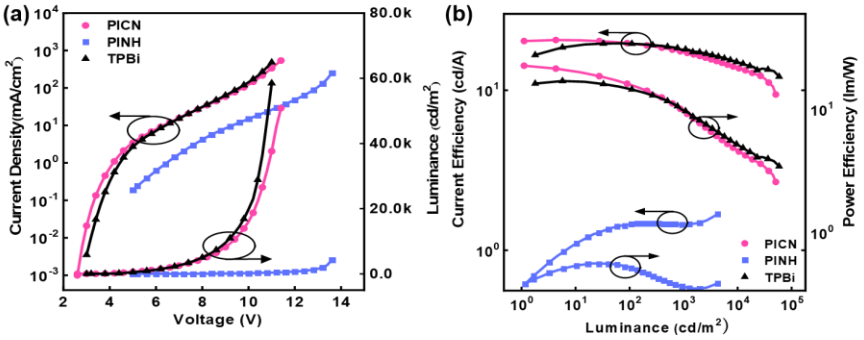

Figure S19. Performances of red PhOLEDs of different ETMs. (a) J-V-L curves, (b) CE-L-PE curves.

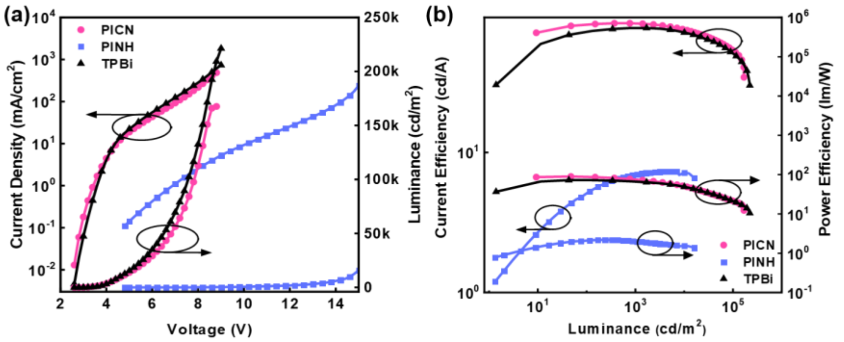

Figure S20. Performances of green PhOLEDs of different ETMs. (a) J-V-L curves, (b) CE-L-PE curves.

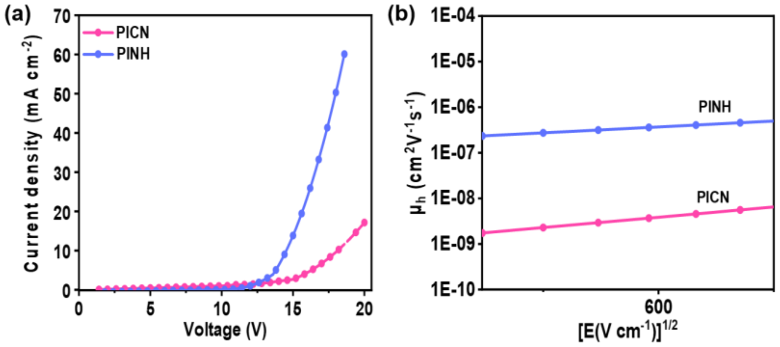

Figure S21. (a) Hole-only J-V curves, (b) Hole mobilities of **PICN** and **PINH** under different electric fields. The configuration of hole-only device: ITO/HATCN (20 nm)/ETM (50 nm)/HATCN (20 nm)/Al (100 nm).

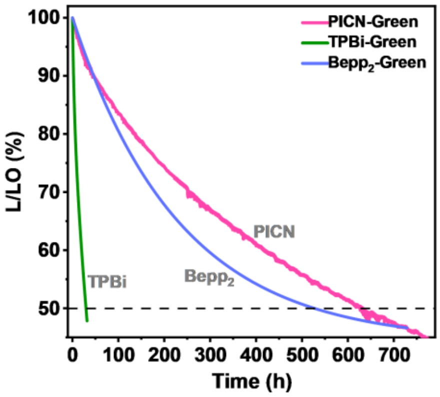

Figure S22. Lifetime curve of the green PhOLED at a fixed current density with an L_0 of 1000 cd/m^2.

Ⅳ. Supplementary Tables

Table S 1. The summary of electrochemical, photophysical and thermal properties of **PICN**, **PINH**.

Molecules	HOMO[a] (eV)	LUMO[b] (eV)	E_g[c] (eV)	E_T[d] (eV)	T_g[e] (°C)	T_m[f] (°C)	T_d[g] (°C)	μ_h [h] [cm²/(v·s)]	μ_{ele} [i] [cm²/(v·s)]
**PICN**	-5.5	-2.6	2.9	2.65	Nd.	240	317	5.24×10^{-9}	1.52×10^{-4}
**PINH**	-5.4	-2.7	2.7	2.70	Nd.	263	335	4.39×10^{-7}	9.70×10^{-7}

[a] HOMO: highest occupied molecular orbital; [b] LUMO: lowest unoccupied molecular orbital; [c] E_g: energy gaps; [d] E_T: triplet state energy level; [e] T_g: glass transition temperatures; [f] T_m: melting temperatures; [g] T_d: temperature of 5% weight loss; [h] [i] Hole and electron mobility under electric field of 4.0×10^5 V/cm.

Table S2. Visualized interactions and combination energy of **PICN** and **PINH** dimers.

	**Visualized Interactions**	**Combination Energy (eV)**
**PICN**	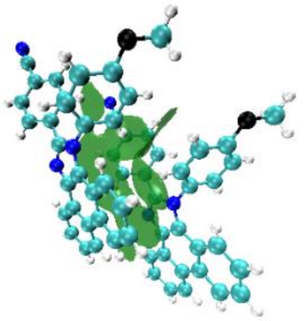	0.889
	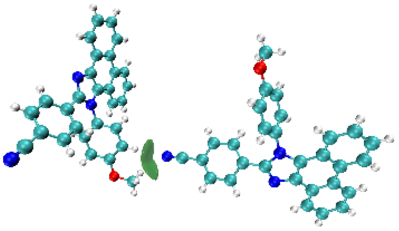	0.207
	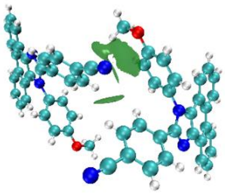	0.220
**PINH**	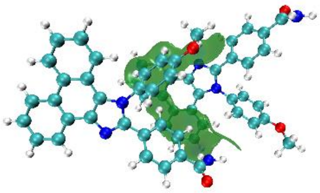	0.477
**PINH**	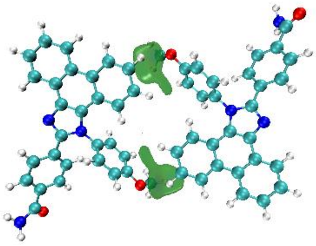	0.355

Table S3. Performances comparison of the two materials and typical ETMs.

ETM	T_g[c] [eV]	E_T[b] [eV]	μ_{ele}[c] @10^5 V/cm [$cm^2/(V\cdot s)$]	Reference
**PICN**	Nd.	2.65	1.52×10^{-4}	This work
**PINH**	Nd.	2.70	9.70×10^{-7}	
**Alq_3**	175	1.52	1.00×10^{-6}	1
**BAlq**	92	2.32	3.10×10^{-5}	2
**$BePP_2$**	Nd.	2.64	4.90×10^{-6}	3
**mNBIPO**	78	2.83	1.70×10^{-7}	4
**TRZ-m-Phen**	120	2.36	5.20×10^{-6}	5
**TPBi**	122	2.67	$10^{-5}\sim10^{-6}$	6
**Bphen**	60	2. 50	5.00×10^{-4}	5
**BCP**	-	2. 60	6.00×10^{-7}	7
**TmPyPB**	79	2. 80	1.00×10^{-3}	8
**Tm4PyPB**	99	2. 80	7.00×10^{-4}	9
**BmPyPB**	-	2. 80	1.00×10^{-4}	10
**3TYMPB**	-	2. 70	1.00×10^{-5}	11
**B4PYPPM**	-	2. 80	1.00×10^{-6}	12
**TemPPB**	97	2. 70	3.00×10^{-7}	13
**DPPyA**	135	2. 50	1.00×10^{-3}	14
**BPBiPA**	185	1. 80	1.00×10^{-3}	15
**T3PySS**	142	1. 80	2.00×10^{-4}	16

[a] Glass transition temperature; [b] Lowest triplet state energy level; [c] Electron mobility at an electric field of 10^5 V/cm.

Table S4. calculated λ-s and the mobilities of **PICN** and **PINH** single crystals.

	λ_{hole}[a](eV)	$\lambda_{electron}$[b] (eV)	μ_h($cm^2\cdot V^{-1}\cdot s^{-1}$)	μ_{ele}($cm^2\cdot V^{-1}\cdot s^{-1}$)
**PICN**	14.823	0.469	~ 0	0.0729
**PINH**	20.168	42.980	~ 0	~ 0

[a] Hole recombination energy; [b] Electron recombination energy.

Table S5. Summary of the OLEDs performances of **PICN**, **PINH** and TPBi.

Emitter		$V_{turn-on}$[a] [V]	EQE[b] [%] **max/ 1000**	CE_{max}[c] [cd/A]	PE_{max}[d] [lm/W]	L_{max}[e] [cd/m^2]	EL[f] [nm]	CIE [g] (x, y)	LT50 [h] [h]
Blue FOLED	PICN	3.0	7.7/6.3	15.7	11.8	83180	466/496	(0.16, 0.31)	330
	TPBi	3.0	7.3/6.2	14.5	9.6	91015	466/497	(0.16, 0.31)	40
	PINH	3.4	0.9/0.9	1.4	1.2	3633	433/466/499	(0.16, 0.20)	NO TEXT
Red PhOLED	PICN	2.8	17.1/13.6	20.6	22.8	50846	622	(0.65, 0.36)	460
	TPBi	3.0	15.9/13.5	19.6	17.1	58465	620	(0.64, 0.36)	98
	PINH	4.8	1.1/1.1	1.7	0.4	4227	431/454/620	(0.48, 0.34)	NO TEXT
Green PhOLED	PICN	2.6	21.9/21.8	83.6	89.6	167649	526	(0.33, 0.63)	630
	TPBi	2.6	20.4/20.3	77.3	72.4	221332	525	(0.32, 0.64)	30
	PINH	5.0	2.1/2.1	7.3	2.2	15955	431/454/525	(0.33, 0.55)	NO TEXT

[a] $V_{turn-on}$: Turn-on voltage at 1 cd/m^2. [b] EQE_{max}/1000: EQE of maximum/at 1000 cd/m^2. [c] CE_{max}: Maximum current efficiency. [d] PE_{max}: Maximum power efficiency. [e] L_{max}: Maximum luminance. [f] EL: EL peak wavelength. [g] CIE: Commission International de L´eclairage recorded at EQE_{max}. [h] LT50: Time to attain 50% of an initial luminance.

Table S6. The single crystal datas of the two molecules

Compound	**PICN**	**PINH**
Chemical formula	C29H19N3O	C29H21N3O2
Formula weight	425.47	886.97
Crystal system	triclinic	triclinic
a/Å	18.684(4)	21.029(12)
b/ Å	6.0072(13)	12.187(7)
c/ Å	19.711(4)	17.975(10)
α/°	90	90
β/°	102.008(4)	105.531(11)
γ/°	90	90
Unit cell volume/ Å3	2163.9(8)	4438(4)
Temperature/K	296 K	296(2)
Space group	P 21/n	P 21/c
Z	4	8
Density (calculated) /g cm^{-3}	1.306	1.327
F(000)	888.0	1856.0
Theta range for data collection	0.997 to 24.999 deg.	2.41 to 21.61 deg.
Reflections measured	13140	27501
Independent reflections	4959	5462
Rint	0.073173	0.170000
Completeness to theta = 72.13°	0.997	0.999
Max. and min. transmission	0.9167 and 0.8860	0.4054 and 0.7456
Goodness-of-fit on F^2	1.035	1.266
Final R_1 values ($I > 2\sigma(I)$)	0.0671	0.0686
Final $wR(F^2)$ values ($I > 2\sigma(I)$)	0.1687	0.1744
Final R_1values (all data)	0.1853	0.1775
Final $wR(F^2)$ values (all data)	0.2399	0.4284
CCDC number	2219823	-

Ⅴ. References

1 G. Jin, J.-Z. Liu, J.-H. Zou, X.-L. Huang, M.-J. He, L. Peng, L.-L. Chen, X.-H. Zhu, J. Peng and Y. Cao, *Sci. Bull.*, 2018, **63**, 446–451.

2 M. Brinkmann, G. Gadret, M. Muccini, C. Taliani, N. Masciocchi and A. Sironi, *J. Am. Chem. Soc.*, 2000, **122**, 5147–5157.

3 S. L. M. Van Mensfoort, R. J. De Vries, V. Shabro, H. P. Loebl, R. A. J. Janssen and R. Coehoorn, *Org. Electron.*, 2010, **11**, 1408–1413.

4 Y. Li, Y. Liu, W. Bu, D. Lu, Y. Wu and Y. Wang, *Chem. Mater.*, 2000, **12**, 2672–2675.

5 B. Wang, X. Lv, B. Pan, J. Tan, J. Jin and L. Wang, *J. Mater. Chem. C*, 2015, **3**, 11192–11201.

6 S.-J. Yeh, M.-F. Wu, C.-T. Chen, Y.-H. Song, Y. Chi, M.-H. Ho, S.-F. Hsu and C. H. Chen, *Adv. Mater.*, 2005, **17**, 285–289.

7 I.-W. Wu, P.-S. Wang, W.-H. Tseng, J.-H. Chang and C.-I. Wu, *Org. Electron.*, 2012, **13**, 13–17.

8 S. Su, T. Chiba, T. Takeda and J. Kido, *Adv.. Mater.*, 2008, **20**, 2125–2130.

9 S. Su, Y. Takahashi, T. Chiba, T. Takeda and J. Kido, *Adv. Funct. Mater.*, 2009, **19**, 1260–1267.

10 S. Su, E. Gonmori, H. Sasabe and J. Kido, *Adv. Mater.*, 2008, **20**, 4189–4194.

11 D. Tanaka, T. Takeda, T. Chiba, S. Watanabe and J. Kido, *Chem. Lett.*, 2007, **36**, 262–263.

12 H. Sasabe, T. Chiba, S.-J. Su, Y.-J. Pu, K. Nakayama and J. Kido, *Chem. Commun.*, 2008, 5821.

13 L. Xiao, B. Qi, X. Xing, L. Zheng, S. Kong, Z. Chen, B. Qu, L. Zhang, Z. Ji and Q. Gong, *J. Mater. Chem.*, 2011, **21**, 19058.

14 D. Zhang, J. Qiao, D. Zhang and L. Duan, *Adv. Mater.*, 2017, **29**, 1702847.

15 D. Zhang, P. Wei, D. Zhang and L. Duan, *ACS Appl. Mater. Interfaces*, 2017, **9**, 19040–19047.

16 *K. Duan, Y. Zhu, Z. Liu, D. Wang, C. Deng, S. Niu, T. Tsuboi, Q. Zhang, Chem. Eng. J*, 2022, **429***, 132215.*
